# Long-Term Follow-Up of Macular Perfusion Evaluated by Optical Coherence Tomography Angiography after Rhegmatogenous Retinal Detachment Surgery

**DOI:** 10.3390/jcm11226725

**Published:** 2022-11-14

**Authors:** Isabel Bartolomé-Sesé, María D. Díaz-Barreda, Elvira Orduna-Hospital, Ana Boned-Murillo, Francisco J. Ascaso, Isabel Pinilla

**Affiliations:** 1 Department of Ophthalmology, Lozano Blesa University Hospital, 50009 Zaragoza, Spain; 2 Aragón Health Research Institute (IIS Aragón), 50009 Zaragoza, Spain; 3 Department of Applied Physics, University of Zaragoza, 50009 Zaragoza, Spain; 4 Department of Surgery, University of Zaragoza, 50009 Zaragoza, Spain

**Keywords:** optical coherence tomography angiography, rhegmatogenous retinal detachment, pars plana vitrectomy, microvascular changes, superficial capillary plexus, deep capillary plexus, choriocapillaris, vessel density, foveal avascular zone

## Abstract

Background: The goal of this study was to investigate macular microvascular changes using optical coherence tomography angiography (OCTA) at one year after successful rhegmatogenous retinal detachment (RRD) surgery. Methods: We performed a cross-section study including RRD treated by pars plana vitrectomy (PPV) with or without scleral buckling and SF6 tamponade. After 12 months, DRI-Triton SS-OCTA was performed. Superficial and deep retinal capillary plexuses (SCP and DCP), choriocapillaris (CC) vessel density (VD), and foveal avascular zone (FAZ) morphology were analyzed. Results were compared with the unaffected contralateral eye. Results: Sixty eyes were included. We observed an increase in VD in the central area of both the SCP and DCP in macula-off eyes treated with PPV + SB and in the SCP of macula-off eyes treated with PPV. Macula-off eyes had a diminished VD for both plexuses in the superior quadrant and in the SCP inferior quadrant in those treated with PPV + SB. The CC flow was diminished in the temporal quadrant of macular-off eyes treated with PPV + SB. Healthy eyes presented higher diameter values than macula-off eyes treated with PPV + SB. FAZ horizontal and vertical diameters were smaller in patients with macula-off RRD vs. macula-on RRD and control groups. Conclusion: Macular vascularity remains almost unchanged one year after successful RRD surgery, irrespective of the surgical technique or prior macular status.

## 1. Introduction

Rhegmatogenous retinal detachment (RRD) is an important health issue, with an incidence of between 9.5 and 18.2 new cases per 100,000 subjects per year and an increasing prevalence [1,2,3,4]. 

RRD surgery has an anatomical success rate of >90%, although visual recovery is not always complete, depending mainly on the macular status and the evolution of the macular detachment [5]. An undetected macular damage at the microstructural level could be responsible for poor vision recovery, changes in color perception, metamorphopsia, and other symptoms [6].

Optical coherence tomography (OCT) allows for a structural evaluation of the retinal anatomy. In RRD, OCT evaluates anatomical changes related to the detached retina such as disruption in the external limiting membrane, changes in the photoreceptor ellipsoid zone, the presence of remnant subretinal fluid, macular edema or epiretinal membranes, and a diminished thickness of the outer nuclear layer [7,8,9].

OCT angiography (OCTA) is a non-invasive, dye-free procedure used to study the retinal capillary plexuses and choriocapillaris (CC) and is based only on the red cell movement inside the vessels [10]. Changes in the capillary vessels could serve as a marker of functional impairment and can correlate with the presence of photoreceptor or other neuron neurodegeneration, as has been described in other retinal diseases with vascular impairment [11]. However, microvascular modifications after RRD surgery are not well-described, and it is not clear if they are associated with unrecovered visual acuity (VA) in otherwise successful RRD surgeries without clear changes on OCT. The fovea is specialized to achieve the best VA; to obtain the finest vision, only some retinal cells are presented in the area, such as photoreceptors, retinal pigment epithelium, and Müller cell bodies. Both the fovea and the foveal slope lack retinal vessels; this area is known as the foveal avascular zone (FAZ) [12]. The FAZ varies in size and shape (acircularity index) across healthy subjects [13], in different retinal [11] or neurodegenerative diseases, [14,15,16,17] and in cardiovascular systemic pathology [18].

The aim of our study was to evaluate the status of both retinal and choroidal capillary plexuses and to characterize the changes in the morphology and size of the FAZ after anatomical-successful RRD surgery, depending on the macular status prior to surgery and the performed procedure, at one year after the intervention.

## 2. Materials and Methods

We performed a retrospective, non-randomized study (2019 to 2020) in the Ophthalmology Department at the Lozano Blesa University Hospital, Zaragoza, Spain. We studied 60 eyes of 57 patients who suffered from a primary RRD that was successfully resolved with a single intervention by 23G pars plana vitrectomy (PPV) with or without scleral buckling (SB) with sulfur hexafluoride (SF6) as tamponade by the same surgeon (IP). The patients that fulfilled the inclusion criteria were recruited. Primary retinal reattachment was obtained in all patients. The study was approved by the Aragon Clinical Research Committee with ID PI18/117. The study adhered to the tenets of the Helsinki Declaration and complies with Spanish legislation in the field of biomedical research and the protection of personal data, Organic Law 3/2018 on the Protection of Personal Data, Basic Law 41/2002 regulating patient autonomy and rights and obligations regarding information and clinical documentation, and Law 14/2007 on biomedical research. Patients signed an Informed Consent form.

Inclusion criteria were the existence of an RRD less than 2 weeks from the onset of the symptoms and surgery and a complete anatomical reparation with a single procedure. Exclusion criteria were traumatic or tractional detachment, failure or impossibility to cooperate in carrying out the examination, proliferative vitreoretinopathy of any grade or previous retinal detachment in the study eye, and any other ocular pathology affecting central vision at the beginning of the study or in the follow-up time (age-related macular degeneration, epiretinal membrane, macular hole or scar, diabetic retinopathy, diabetic macular edema, IOP over 22 mmHg, diagnosed glaucoma, or amblyopia). 

The control group consisted of 52 contralateral healthy eyes.

**Figure 1:**
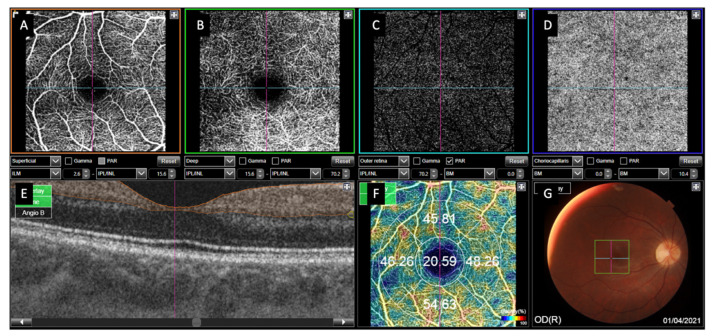
Image of 3 × 3 mm optical coherence tomography angiography (OCTA) performed by Deep-Range Imaging (DRI)-Triton SS-OCT device. (**A**) Retinal superficial capillary plexus (SCP). (**B**) Retinal deep capillary plexus (DCP). (**C**) Outer retina. (**D**) Choriocapillaris plexus (CC). (**E**) OCT profile (in orange the area in which SCP vessel density (VD) is studied). (**F**) Vessel density at the SCP as the percentage of pixels occupied by blood flow in the central area and in the 4 quadrants. (**G**) Fundus photography showing the examined OCTA area as a square in the central area.

All patients underwent a thorough medical history examination at the first visit, including age, sex, family history of ophthalmological and systemic diseases, and personal medical and surgical history, including medication. The date of symptom onset and intervention, as well as the macular status at the time of diagnosis of RRD, were collected. Macular status prior to surgery was checked using Spectralis spectral domain ODT (SD-OCT) (Spectralis^®^, Heidelberg Engineering, Inc., Heidelberg, Germany). RRDs were divided into 2 subgroups according to the macular involvement: macula-on RRD and macula-off RRD. The minimum follow-up time was one year. A complete ophthalmological examination was performed at all visits before and after surgery. This included spherical equivalent (SE) and axial length (AL) measured with the Aladdin KR-1W Series optical biometry system (Topcon Corporation, Tokyo, Japan); best-corrected VA (BCVA) measured with Snellen charts converted in the minimal angle of resolution (log MAR); intraocular pressure (IOP) measured by Goldmann tonometry applanation and slit-lamp examination; and fundus examination by Spectralis OCT, swept-source OCT (SS-OCT), and Deep-Range Imaging (DRI)-Triton SS-OCT (DRI)-Triton^®^, Topcon Corporation, Tokyo, Japan). OCTA was performed using DRI-Triton SS-OCT after pupil dilatation with Tropicamide^®^ (Alcon Cusi, Barcelona, Spain). A macular three-dimensional scan (size 6 × 6 mm) and a 3 × 3 mm OCTA were obtained with IMAGEnet 6 software Version 1.22.1.14101^©^ 2014 (Topcon Corporation, Tokyo, Japan). VD was provided by the device for both retinal plexuses, the superficial capillary plexus (SCP), and the deep capillary plexus (DCP) and CC (Figure 1). 

**Figure 2:**
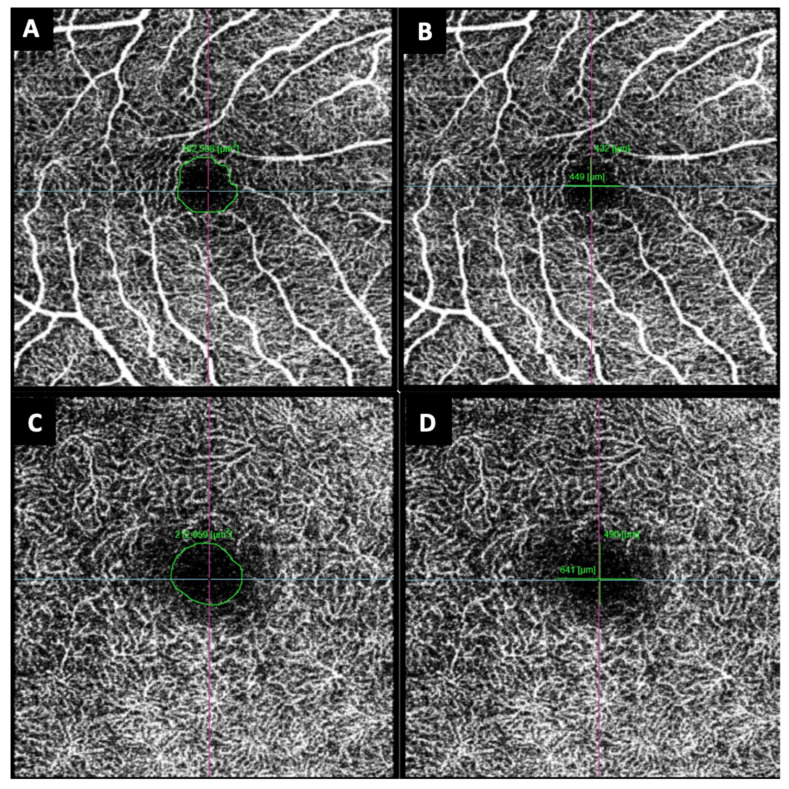
Example of foveal avascular zone (FAZ) area and diameters measured manually in both superficial (SCP) and deep capillary retinal plexuses (DCP). (**A**) FAZ area of the SCP. (**B**) FAZ diameter of the SCP. (**C**) FAZ area of the DCP. (**D**) FAZ diameter of the DCP.

The FAZ area and the horizontal and vertical diameters of both the SCP and DCP were measured manually with the measurement tool provided by the device. Measurements were taken by two independent investigators (IB, IP) and the media of both values were considered (Figure 2).

Statistical analysis was performed using the Statistical Package for the Social Sciences (SPSS 25.0, SPSS, IBM, Armonk, NY, USA). Variables were tested for normality using the Kolmogorov–Smirnov test. Depending on whether the variables were normal or not, comparisons were made using Student’s *t* test for independent samples or the Mann–Whitney U test, respectively. These comparisons were between macula-on RRD and macula-off RRD with the contralateral eye in each case, being a controlled study. A *p* value < 0.05 was considered statistically significant.

## 3. Results

**Figure 3:**
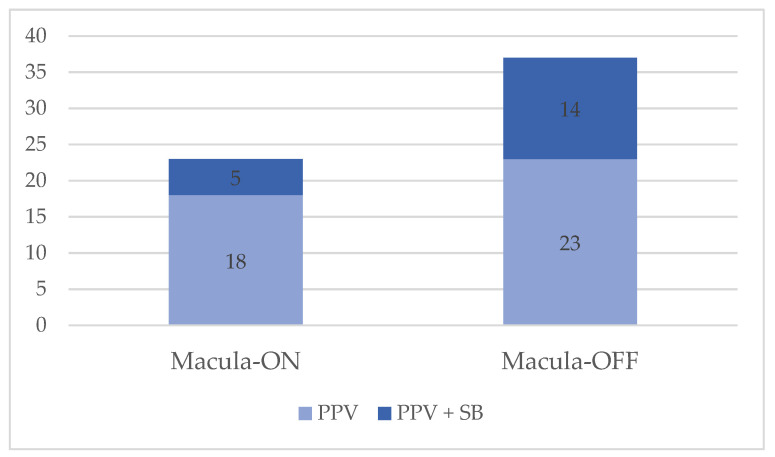
Macular status prior to surgery and type of surgery. Abbreviations: PPV, pars plana vitrectomy; SB, scleral buckling.

Clinical and demographic characteristics of the RDD:

A total of 60 eyes of 57 patients were studied. The mean age was 59.14 ± 9.52 years; 73.7% of the patients were male and 26.3% were female. Three of the males had a contralateral RRD in the follow-up time. Forty-one eyes underwent PPV alone (68.3%), whereas 19 eyes underwent PPV + SB (31.7%). The group was formed by a total of 23 eyes (38.3%) with macula-on RRD and 37 (61.7%) with macula-off RRD. In the macula-off RRD group, twenty-three eyes were treated only with PPV (62.1%) and fourteen with PPV + SB (37.8%); in the macula-on RRD group, five eyes underwent a PPV + SB (21.7%). Twenty eyes (33.3%) had a previous history of cataract surgery in the affected eye. The mean RRD duration from diagnosis to the date of surgery was 4.75 ± 2.55 days, while the duration of symptoms to the diagnosis was 4.67 ± 5.23 days. Demographics and clinical characteristics are described in Table 1 and Figure 3.

We observed no differences in VA, refractive error (RE), and AL between eyes affected by macula-on RRD vs. healthy fellow eyes and eyes with macula-off RRD. We found differences in VA and RE between macula-off eyes and control eyes. Eyes with RRD had greater myopic RE with no differences in AL between affected and unaffected eyes.

OCTA VD findings:

In order to evaluate the influence of the performed surgical technique in the OCTA findings, RRD was divided in two subgroups: RRD treated with PPV and RRD treated with PPV + SB. We also evaluated the macular status prior to surgery to assess any relationship with retinal and choroidal flow, dividing the studied eyes into macula-on RRD and macula-off RRD groups. OCTA VD characteristics in eyes with RRD and the fellow healthy eyes are summarized in Table 2. 

We observed no differences in VD within the control group and the macula-on RRD group without influence of the different surgical techniques. 

Treated macula-off RRD eyes showed differences with the fellow healthy eyes. The central area of the SCP had a significative flow increase in the macula-off RRD eyes treated with PPV + SB vs. the untreated eyes (26.73 ± 7.9 vs. 21.08 ± 4.74, respectively; *p* = 0.001). We also found an increased flow in the central area of the DCP in treated macula-off RRD eyes for both surgical procedures (macula-off RRD treated with PPV 26.44 ± 5.92, macula-off RRD treated with PPV + SB 29.57 ± 9.76, untreated eyes 21.25 ± 56.55; *p* < 0.001 and <0.01, respectively). However, vertical quadrants had a diminished VD in treated macula-off RRD eyes vs. fellow eyes. The superior quadrant had a lower VD in both SCP and DCP for both surgical techniques (SCP: macula-off RRD eyes treated with PPV 46.39 ± 4.26, macula-off RRD eyes treated with PPV + SB 45.18 ± 4.66, fellow eyes 49.3 ± 4.01; *p* = 0.006 and *p* = 0.002, respectively. DCP: macula-off RRD eyes treated with PPV 48.01 ± 3.99, macula-off RRD eyes treated with PPV + SB 48.10 ± 3.91, fellow eyes 51.06 ± 4.21; *p* = 0.005 and *p* = 0.021, respectively). In the inferior quadrant, we only found differences when comparing the SCP flow between macula-off RRD eyes treated with PPV + SB (46.4 ± 3.91) vs. fellow eyes (50.23 ± 3.99); *p* = 0.02 (Table 2). 

Looking for changes in the CC flow, we only found a diminished temporal flow in macula-off RRD eyes treated with PPV + SB compared not only with control group, but also macula-off RRD eyes treated with PPV alone (macula-off RRD eyes treated with PPV + SB 51.99 ± 2.51; macula-off RRD eyes treated with PPV 53.87 ± 2.40; control eyes 53.57 ± 2.41; macula-off RRD eyes treated with PPV vs. PPV + SB *p* = 0.03; and fellow eyes vs. PPV + SB *p* = 0.036) (Table 2).

FAZ evaluation:

We evaluated the mean value of the FAZ area and both horizontal and vertical diameters of the SCP and DCP. The diameters were evaluated to assess FAZ morphology and circularity. In the FAZ area, we did not find size differences between the macula-on RRD group and the control eyes, irrespective of which surgical technique was performed. However, we found a statistically significant smaller area in those eyes with macular involvement treated with PPV + SB vs. the control group in both SCP and DCP. Macula-off RRD eyes treated with PPV showed no differences vs. the control group or the macula-on RRD group. The results are presented in Table 3. 

Both horizontal and vertical FAZ diameters in the macula-off RRD group were statistically significantly smaller than both the macula-on RRD group and the control group in both SCP and DCP. The FAZ diameters were similar in healthy eyes and in patients with macula-on RDD. At one year after surgery, we found statistically significant higher diameter values for healthy eyes than macula-off RDD eyes in both SCP and DCP (horizontal values of the SCP were 473.25 vs. 403.11; *p* = 0.03 and DCP 443.37 vs. 365.54; *p* = 0.031 in healthy eyes and macula-off RRD eyes, respectively). The vertical diameters were 468.62 vs. 400.81 in the SCP (no significative difference; *p* = 0.05) and 476.37.30 vs. 392.11 (*p* = 0.021) in the DCP in healthy eyes and macula-off RRD eyes, respectively. Patients with macula-on RRD presented significantly higher FAZ diameters at one year after surgery than those patients with macula-off RRD. Looking for differences between surgical techniques, we saw that patients with macula-off RRD that underwent PPV + SB generated differences in both the FAZ area and the diameters.

**Figure 4:**
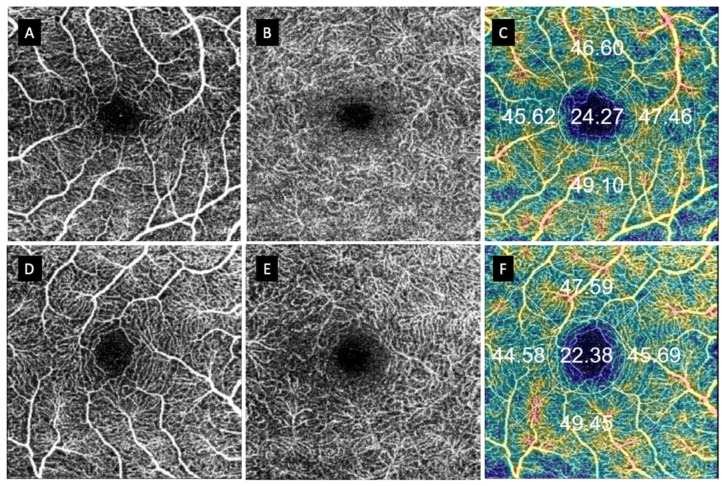
Anatomical finding in patients with macula-on RDD vs. their fellow eyes. (**A**–**C**) Macula-on RDD. (**D**–**F**) Fellow healthy eye. (**A**,**D**) represent superficial capillary plexus (SCP); (**B**,**E**) represent deep capillary plexus (DCP); (**C**,**F**) represent VD in the SCP.

**Figure 5:**
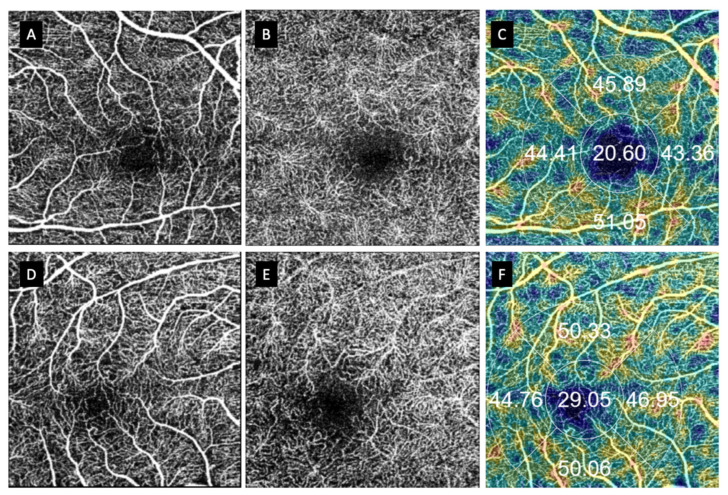
Anatomical finding in patients with macula-off RDD vs. their fellow eyes. (**A**–**C**) Macula-on RDD. (**D**–**F**) Contralateral healthy eye. (**A**,**D**) represent superficial capillary plexus (SCP); (**B**,**E**) represents deep capillary plexus; and (**C**,**F**) represents vessel density in the SCP.

Looking for changes in the FAZ circularity, we calculated the difference between the vertical and horizontal diameter in the FAZ of both retinal plexuses. We used their absolute values to assess the symmetry or circularity of the FAZ. It is considered that in a perfect circle, both diameters should be equal. Therefore, the greater the difference between both diameters, the greater the acircularity of the FAZ. We only found differences in the mean diameter value in the DCP between the macula-off RRD eyes treated with PPV and healthy eyes (*p* = 0.036), assuming that the acircularity was greater in this RDD group (104.91 vs. 66.23; *p* = 0.036) (Table 4). Although we were not able to find any differences, we found some FAZ abnormalities in patients with macula-off RDD eyes vs. healthy fellow eyes that were difficult to grade (Figure 4 and Figure 5).

## 4. Discussion

Visual recovery after RRD surgery is not always complete, and although there are factors related to visual recovery, there are still unknown factors that can modify functional improvement [19]. In our study, we checked microvascular modifications in 60 eyes after successful RRD surgery, focusing on the previous status of the macula. OCTA gives us detailed information on the capillary status in the retina and choroid and their changes after treatment. We looked for long-term alterations at one year after the surgery. We postulated that there might be changes in macular microvascularization that could be related to the changes in the retinal cells and may be responsible for the incomplete visual recovery in patients with RRD. It is known that there is great variability among subjects in both the FAZ and VD. To avoid this variability, we studied the contralateral eye as the control [20]. In studies that do not use the contralateral eye as the control, different authors have found differences that could be only attributed to interindividual differences.

In our patients, VD in the macular area and FAZ remained unchanged in patients with macula-on RRD, regardless of the performed treatment (PPV with or without SB). Similar to our findings, Yoshikawa et al. showed no changes in the FAZ area and in VD in five eyes with macula-on RRD [21]. Woo et al. and Resch et al., in 15 and 20 patients, respectively, showed no differences in VD between macula-on RRD eyes vs. the healthy fellow eyes, or control group [22,23]. Barca et al. revealed a decrease in VD in eyes without macular involvement vs. healthy eyes during the first months after surgery with gradual recovery; the differences disappeared six months after surgery [24]. However, Bonfiglio et al., [25] following 56 patients’ macula-on RDD for 12 months, found a decrease in parafoveal DCP with no differences in the FAZ area.

In the macula-off RRD group, we also did not find differences between both subgroups, irrespective of the treatment received in each case. However, significant differences were found when each treatment group was compared with fellow healthy eyes. We found an increase in the mean VD in the central area in both the SCP and DCP. Nevertheless, we found a diminution in VD in both vertical quadrants and both retinal plexuses. A decrease in VD in the CC was observed only in the temporal field in eyes that underwent PPV + SB (vs. those that were only treated with PPV and their respective fellow eye). Although it is difficult to explain this VD increase, in the central area of both retinal plexuses, we could postulate that there is a modification in the capillary retinal vascularization related to the modification of the foveal structure. Our macula-off RRD group had a short evolution time and a good VA recovery (LogMAR VA 0.203 ± 0.16). Some authors, such as Ng and coworkers, correlate VA with a smaller FAZ; in their 12-month follow-up study, they found that those patients with a smaller FAZ after macula-off RRD treatment were the ones with better VA recovery, probably related to an angiogenesis stimulation in the DCP after the macular detachment trying to compensate for the damage or a retinal contraction; however, they did not find differences in the FAZ area between groups. Bonfiglio et al., [25] in their macula-off RRD patients (*n* = 37), found no FAZ differences, but a diminished parafoveal SCP and foveal and parafoveal DVP. Nam et al. conducted a study with 34 patients and found that recovery of macular vascularization in eyes treated with PPV was lower than in those treated with SB [26]. Tsen et al. [27] evaluated 28 eyes after macula-off RRD treatment: 11 eyes underwent PPV alone, and 12 eyes were treated with PPV + SB. Evaluating all the eyes without considering the surgical technique, they found a diminished mean VD and parafoveal VD in both the SCP and DCP. A higher VD was observed in the CC of those eyes that were treated with PPV alone. Unlike our results, eyes treated with PPV + SB were the ones with lower VD. They extended the evaluation only to the first three postoperative months; in our series, with one year of follow-up, we can postulate a gradual recovery of the macular microcirculation. Another explanation could be an increase in vascular congestion induced by the photoreceptor ischemia related to the detachment. Supporting this theory, Cardillo Piccolino [28] described a capillary dilatation and hyperpermeability (in response to tissue hypoxia) in 50 eyes studied with fluorescein angiography. Lu et al., studying 31 macula-off RDDs, found no differences in SCP and DCP VD vs. their fellow eyes. They showed a diminution in peripapilar VD with a correlation to VA recovery [29]. We were not able to find differences in VD dependent on the surgical procedure; the only CC VD diminution that we showed was in the combined procedure, which could be related to other factors difficult to measure, including perfluorocarbon liquid injection and any increase in IOP (among others); we did not find any change in retinal vascularization related to those factors. 

Resch and coworkers studied OCTA findings over long-duration follow-up periods (6–12 months, 1–2 years, and 2–10 years). They used different endotamponades including SF6, C3F8, and silicone oil. Like our study, they did not find differences between macula-on RRD and healthy eyes. They found a reduction of the VD in RRD eyes with no recovery during follow-up; this reduction was mainly in the DCP in the initial periods of follow-up. In long-term follow-up eyes, the SCP was more affected, and the area of non-perfusion increased [11].

Reduction of VD and enlargement of the FAZ area has been reported by some authors, based mainly in the previous macular status. Aniruddha et al. showed a significant increase in the FAZ area and a reduction of VD and fractal dimension, which assesses the complexity of the vascular microarchitecture [30]. McKay et al., in 17 RRD macula-off eyes, detected a diminished VD in the DCP with no changes in the FAZ [31]. Hassanpoor et al. [32] studied 24 RRD eyes successfully treated with 360° encircling SB; unlike the outcomes in our research, they obtained a lower VD in the parafoveal area, with no differences in the central area. Wang et al. [33] studied retinal microcirculation in the first three months after surgery; they observed a progressive increase in VD, with a gradual recovery of macular perfusion and no difference in the contralateral healthy eye at the end of the follow-up. They suggested a rehabilitation curve of the different retinal plexuses, with gradual macular vascularization recovery with the attached and flattened retina. Çetinkaya-Yaprak et al., also in the first 3 months after surgery, demonstrated an increase in the FAZ area and a diminished retinochoroidal flow [34]. 

The FAZ has a rounded or oval morphology in healthy eyes and may be altered in those who have vascular pathologies, changing the typical round shape of this structure. The circularity index (CI) is defined as the correlation between the perimeter of the FAZ and the circumference [35]. To our knowledge, there are no studies that have analyzed this parameter in patients with RRD. However, FAZ circularity has been analyzed in other retinal pathologies, including diabetic retinopathy or epiretinal membrane (ERM) [11,36]. Hirata et al. evaluated the FAZ area and perimeter in patients who underwent PPV due to ERM and found them significantly smaller than in the contralateral eye during the 12-month follow-up, and both parameters and their interocular ratios related to the postoperative aniseikonia [36]. In our study, we analyzed the difference between the horizontal and vertical diameter to evaluate the CI, considering that in a circumference, this must be zero. In our study, the FAZ diameters followed a peculiar pattern: macula-on RRD eyes had higher values than the control group, but macula-off RRD eyes were the ones with smaller sizes. Macular involvement, either by a macula-off RRD or other pathologies such as ERM, could stimulate vascularization in the periphery of the FAZ, reducing its size and altering its CI. We showed a diminished VD in the vertical diameters, suggesting a change in the vascularity pattern of the macular area.

There are a few studies looking at OCTA changes after retinal surgery for RRD using silicone oil as a tamponade. A common finding among those available is retinal thinning, but not all of them found changes in the VD. Lee et al. found an increase in the FAZ and a decrease in VD in the DCP [37]. Ma et al. found a reduction in the SCP, but no changes in the DCP [38], suggesting that the SCP was associated with ganglion cell damage. However, Xiang et al., despite showing a diminished retinal thickness, did not find changes in the FAZ area or VD in both retinal plexuses [39]. Bayraktar et al. showed changes in VD only in their macula-off RRD group [40]. Maqsood et al. [41] observed no significant difference in the FAZ between macula-off RRD eyes and healthy eyes at 12 weeks after the intervention.

Limitations included the number of studied eyes and the different subgroups undergoing only a small number of combined procedures. 

Changes in macular and choroidal microvascularization after successful repairment of RRD are not clear. Despite the fact that there is visual alteration in eyes that have undergone RRD, we were not able to find specific OCTA biomarkers, and the patient number did not allow us to make different subgroups related to VA. The inability to check for changes that can predict functional recovery remains an important issue for retinal surgeons.

## 5. Conclusions

In conclusion, the findings of our study suggest that macular vasculature remains unchanged at one year after successful RRD surgery. Macula-off RRD eyes show changes in the retinal capillary plexuses in terms of blood flow distribution, with changes in the FAZ diameters related to an increase in the FAZ VD but with a diminished flow in the vertical quadrants. The performed surgery (PPV or PPV + SB) had a small influence in retinal and choroidal capillary blood flow. A higher number of patients should be studied to assess our results.

## Figures and Tables

**Table 1:** Clinical and demographic characteristics of the RDD patients. Control group included the fellow healthy eye of the RRD eye.

Sex		Male	Female
*n* = 57		42 (73.7%)	15 (26.3%)
Studied RRD eye		Right eye	Left eye
*n* = 60		26 (43.3%)	34 (56.7%)
Mean age ± SD (years)	59.14 ± 9.52		
Refractive error (D)	−2.89 ± 4.01		
Axial length (mm)	25.74 ± 3.45		
Duration of symptoms (days)	4.75 ± 2.55		
Lens status (%)		Phakic	Pseudophakic
		40 (66.7%)	20 (33.3%)
	Macula-on	Macula-off	Fellow eye
VA (log MAR)	0.143 ± 0.25	0.203 ± 0.16	0.063 ± 0.14
Refractive error	−2.34 ± 3.01	−4.37 ± 5.18	−2.18 ± 0.49
Axial length (mm)	25.43 ± 1.48	26.03 ± 4.35	25.70 ± 3.48

Abbreviations: VA, visual acuity; RRD, rhegmatogenous retinal detachment; SD, standard deviation; D, diopters.

**Table 2:** Vessel density (VD) in superficial and deep retina capillary plexuses (SCP, DCP) and choriocapillaris plexus (CC) measured with Deep-Range Imaging (DRI)-Triton OCTA in macula-on RRD and macula-off RRD eyes, depending on the surgical procedure, and control eyes.

	Macula-On RRD PPV (*n* = 18)	Macula-On RRD PPV +SB (*n* = 5)	Macula-Off RRD PPV (*n* = 23)	Macula-Off RRD PPV + SB (*n* = 14)	Fellow Eye (*n* = 52)	P1 (ON PPV vs. PPV + SB)	P2 (ON PPV vs. Fellow Eye)	P3 (ON PPV + SB vs. Fellow Eye)	P4 (OFF PPV vs. PPV + SB)	P5 (OFF PPV vs. Fellow Eye)	P6 (OFF PPV + SB vs. Fellow Eye)
**Mean central VD**											
SCP	20.57 ± 3.69	20.94 ± 2.47	22.94 ± 4.48	26.73 ± 7.9	21.08 ± 4.74	0.419	0.685	0.950	0.070	0.116	**0.001**
DCP	24.23 ± 5.91	21.34 ± 2.26	26.44 ± 5.92	29.57 ± 9.76	21.25 ± 5.65	0.304	0.067	0.973	0.230	**<0.001**	**<0.01**
CC	48.77 ± 3.27	51.12 ± 3.03	48.26 ± 4.89	47.98 ± 4.33	49.58 ± 3.55	0.164	0.401	0.351	0.860	0.194	0.159
**Mean superior VD**											
SCP	48.16 ± 3.89	47.39 ± 3.33	46.39 ± 4.26	45.18 ± 4.66	49.3 ± 4.01	0.345	0.782	0.309	0.421	**0.006**	**0.002**
DCP	50.49 ± 4.85	49.27 ± 3.38	48.01 ± 3.99	48.10 ± 3.91	51.06 ± 4.21	0.484	0.981	0.364	0.951	**0.005**	**0.021**
CC	51.26 ± 2.35	50.79 ± 1.84	51.13 ± 3.67	52.22 ± 2.52	52.38 ± 2.75	0.341	0.130	0.212	0.339	0.109	0.843
**Mean temporal VD**											
SCP	47.18 ± 4.19	46.07 ± 1.33	46.24 ± 2.98	45.28 ± 3.03	47.04 ± 3.14	0.286	0.885	0.499	0.352	0.308	0.066
DCP	48.16 ± 3.46	48.53 ± 2.34	48.77 ± 3.96	45.32 ± 8.7	47.5 ± 3.54	0.974	0.275	0.530	0.107	0.170	0.155
CC	53.14 ± 1.94	52.49 ± 1.54	53.87 ± 2.40	51.99 ± 2.51	53.57 ± 2.41	0.499	0.500	0.333	**0.030**	0.616	**0.036**
**Mean nasal VD**											
SCP	45.92 ± 3.11	46.08 ± 2.67	45.37 ± 3.5	45.64 ± 3.94	46.55 ± 2.96	0.460	0.449	0.735	0.830	0.139	0.350
DCP	49.63 ± 3.63	48.03 ± 4.43	49.49 ± 5.07	48.86 ± 3.38	49.08 ± 4.09	0.389	0.579	0.587	0.683	0.716	0.848
CC	52.81 ± 2.36	51.99 ± 3.01	52.86 ± 3.68	53.94 ± 2.17	53.10 ± 2.50	0.528	0.667	0.357	0.326	0.744	0.255
**Mean inferior VD**											
SCP	49.39 ± 3.65	49.23 ± 3.86	48.44 ± 6.55	46.41 ± 3.91	50.23 ± 3.99	0.466	0.435	0.594	0.304	0.148	**0.002**
DCP	53.38 ± 3.43	52.17 ± 4.51	50.69 ± 5.43	49.48 ± 4.25	52.01 ± 4.03	0.467	0.159	0.937	0.483	0.245	0.043
CC	53.65 ± 2.46	53.68 ± 1.61	52.58 ± 2.74	52.90 ± 2.83	53.24 ± 3.07	0.981	0.612	0.757	0.732	0.377	0.711

Abbreviations: p, *p* value. P1. Macula-on RRD eyes treated with PPV vs. PPV + SB; P2. Macula-on RRD eyes treated with PPV vs. fellow eye; P3. Macula-on RRD eyes treated with PPV + SB vs. fellow eyes; P4. Macula-off RRD eyes treated with PPV vs. PPV + SB; P5. Macula-off RRD eyes treated with PPV vs. fellow eyes; P6. Macula-off RRD eyes treated with PPV + SB vs. fellow eyes. Differences that reached statistically significance are presented in bold (*p* < 0.05).

**Table 3:** Foveal avascular zone area (FAZ) in mm^2^ and diameters in mm measured in both retinal capillary plexuses; eyes that underwent retinal surgery for rhegmatogenous retinal detachment vs. control eyes.

	FAZ SCP Area	FAZ SCP Horizontal Diameter	FAZ SCP Vertical Diameter	FAZ DCP Area	FAZ DCP Horizontal Diameter	FAZ DCP Vertical Diameter
Macula-on RRD (*n* = 23)	252.77 ± 88.11	499.22 ± 123.664	505.48 ± 119.591	201.255 ± 72.43	448.30 ± 124.832	487.30 ± 141.027
Macula-off RRD (*n* = 37)	210.183 ± 171.254	403.11 ± 168.168	400.81 ± 176.891	170.501 ± 103.475	365.54 ± 150.155	392.11 ± 156.984
Control eye (*n* = 52)	244.204 ± 98.910	473.25 ± 132.170	468.62 ± 147.188	207.26 ± 76.42	443.37 ± 110.114	476.37 ± 117.212
P1 (on vs. off)	0.063	**0.021**	**0.015**	0.218	**0.031**	**0.021**
P2 (on vs. control)	0.765	0.426	0.295	0.751	0.864	0.727
P3 (off vs. control)	0.053	**0.030**	0.05	0.057	**0.006**	**0.005**
Macula-on RRD treated with PPV (*n* = 18)	261.57 ± 90.47	508.72 ± 130.835	504.72 ± 116.695	208.65 ± 72.67	444.61 ± 132.913	487.67 ± 148.558
Macula-on RRD treated with PPV + SB (*n* = 5)	221.097 ± 79.43	465.00 ± 97.414	508.20 ± 144.131	174.60 ± 72.64	461.60 ± 101.704	486.00 ± 124.856
Macula-off RRD treated with PPV (*n* = 23)	238.56 ± 191.85	425.52 ± 154.78	427.87 ± 170.508	191.75 ± 100.012	387.87 ± 103.778	427.74 ± 145.772
Macula-off RRD treated with PPV + SB (*n* = 14)	244.204 ± 123.109	366.29 ± 188.210	356.36 ± 184.462	135.58 ± 103.012	328.86 ± 204.645	333.57 ± 162.320
P1 (on PPV vs. on PPV + SB)	0.333	0.497	0.956	0.376	0.795	0.982
P2 (on PPV vs. control)	0.528	0.329	0.350	0.946	0.969	0.744
P3 (on PPV + SB vs. control)	0.574	0.893	0.567	0.364	0.724	0.862
P4 (off PPV vs. off PPV + SB)	0.210	0.305	0.238	0.110	0.252	0.076
P5 (off PPV vs. control)	0.280	0.176	0.296	0.465	**0.044**	0.129
P6 (off PPV + SB vs. control)	**0.030**	**0.017**	**0.019**	**0.005**	**0.006**	**<0.0001**

Abbreviations: SCP, superficial capillary plexus; DCP, deep capillary plexus. Differences that reached statistically significance are presented in bold (*p* < 0.05).

**Table 4:** Differences of diameters of the FAZ area (in mm^2^) in both the SCP and DCP vs. control eyes depending on the prior macular status and the surgical procedure. Bold values are statistically significant (*p* < 0.05) (Mann–Whitney U test for non-parametric values).

	Patient Number	Difference in SCP Diameter	Difference in DCP Diameter
Macula-on RRD treated with PPV	18	47.20 ± 50.08	55.20 ± 36.45
Macula-on RRD treated with PPV + SB	5	63.22 ± 59.85	107.50 ± 77.22
Macula-off RRD treated with PPV	23	39.30 ± 29.63	104.91 ± 89.97
Macula-off RRD treated with PPV + SB	14	74.07 ± 75.54	84.85 ± 69.17
Control eye	52	57.51 ± 47.67	66.23 ± 55.27
P1 (on PPV vs. on PPV +SB)		0.478	0.205
P2 (on PPV vs. control)		0.154	0.605
P3 (on PPV + SB vs. control)		0.632	0.902
P4 (off PPV vs. off PPV + SB)		0.621	0.442
P5 (off PPV vs. control)		0.925	**0.036**
P6 (off PPV + SB vs. control)		0.154	0.125

P1. Macula-on RRD treated with PPV vs. macula-on PPV + SB; P2. Macula-off RRD treated with PPV vs. macula-off RRD PPV + SB; P3. Macula-on RRD treated with PPV + SB vs. fellow eye; P4. Macula-off RRD treated with PPV + SB vs. fellow eye. P5. Macula-on RRD treated with PPV vs. fellow eye; P6. Macula-off RRD treated with PPV vs. fellow eye. Bold values are statistically significant (*p* < 0.05) (Mann–Whitney U test for non-parametric values).

## Data Availability

Not applicable.
